# Orthology confers intron position conservation

**DOI:** 10.1186/1471-2164-11-412

**Published:** 2010-07-02

**Authors:** Anna Henricson, Kristoffer Forslund, Erik LL Sonnhammer

**Affiliations:** 1 Department of Cell and Molecular Biology, Karolinska Institutet, SE-171 77 Stockholm, Sweden; 2 Stockholm Bioinformatics Centre, Albanova, Stockholm University, SE-106 91 Stockholm, Sweden

## Abstract

**Background:**

With the wealth of genomic data available it has become increasingly important to assign putative protein function through functional transfer between orthologs. Therefore, correct elucidation of the evolutionary relationships among genes is a critical task, and attempts should be made to further improve the phylogenetic inference by adding relevant discriminating features. It has been shown that introns can maintain their position over long evolutionary timescales. For this reason, it could be possible to use conservation of intron positions as a discriminating factor when assigning orthology. Therefore, we wanted to investigate whether orthologs have a higher degree of intron position conservation (IPC) compared to non-orthologous sequences that are equally similar in sequence.

**Results:**

To this end, we developed a new score for IPC and applied it to ortholog groups between human and six other species. For comparison, we also gathered the closest non-orthologs, meaning sequences close in sequence space, yet falling just outside the ortholog cluster. We found that ortholog-ortholog gene pairs on average have a significantly higher degree of IPC compared to ortholog-closest non-ortholog pairs. Also pairs of inparalogs were found to have a higher IPC score than inparalog-closest non-inparalog pairs. We verified that these differences can not simply be attributed to the generally higher sequence identity of the ortholog-ortholog and the inparalog-inparalog pairs.

Furthermore, we analyzed the agreement between IPC score and the ortholog score assigned by the InParanoid algorithm, and found that it was consistently high for all species comparisons. In a minority of cases, the IPC and InParanoid score ranked inparalogs differently. These represent cases where sequence and intron position divergence are discordant. We further analyzed the discordant clusters to identify any possible preference for protein functions by looking for enriched GO terms and Pfam protein domains. They were enriched for functions important for multicellularity, which implies a connection between shifts in intronic structure and the origin of multicellularity.

**Conclusions:**

We conclude that orthologous genes tend to have more conserved intron positions compared to non-orthologous genes. As a consequence, our IPC score is useful as an additional discriminating factor when assigning orthology.

## Background

Assigning function to protein coding genes is one of the most important tasks in the post-genome era. With the wealth of genomes available, automatic methods for identifying evolutionary relationships between genes becomes important when transferring functions from already annotated genes to unannotated. Consequently, it is of the outermost importance that the evolutionary relationships inferred between genes reflects their true evolutionary history. The term "homology" is simply not sufficiently well-defined when describing the evolutionary relationship between genes, and therefore previous publications have established more precise definitions. Orthologs are genes that derive from a single gene in the last common ancestor and have been separated by a speciation event [1]. They can typically be considered as functional counterparts in different species. Paralogs, on the other hand, are genes that derive from a single gene that has been duplicated within a genome. When a gene has been duplicated, one of the copies could potentially be more free to adapt to new functions, whereas the other retains the original function. Paralogs can be further separated into two different subgroups, namely inparalogs and outparalogs, depending on when during evolution the duplication occurred [2]. If the duplication occurred after the speciation event, the genes are considered to be inparalogs, meaning that they are co-orthologs to one or several genes in another species. Analysis of inparalogs can be used to detect lineage-specific adaptations. However, if the duplication event happened prior to the speciation event, the sequences are outparalogs and as such do not form any co-ortholog relationship with genes in another genome. Hence, outparalogs cannot be used to transfer functional assignments between species.

**Figure 1:**
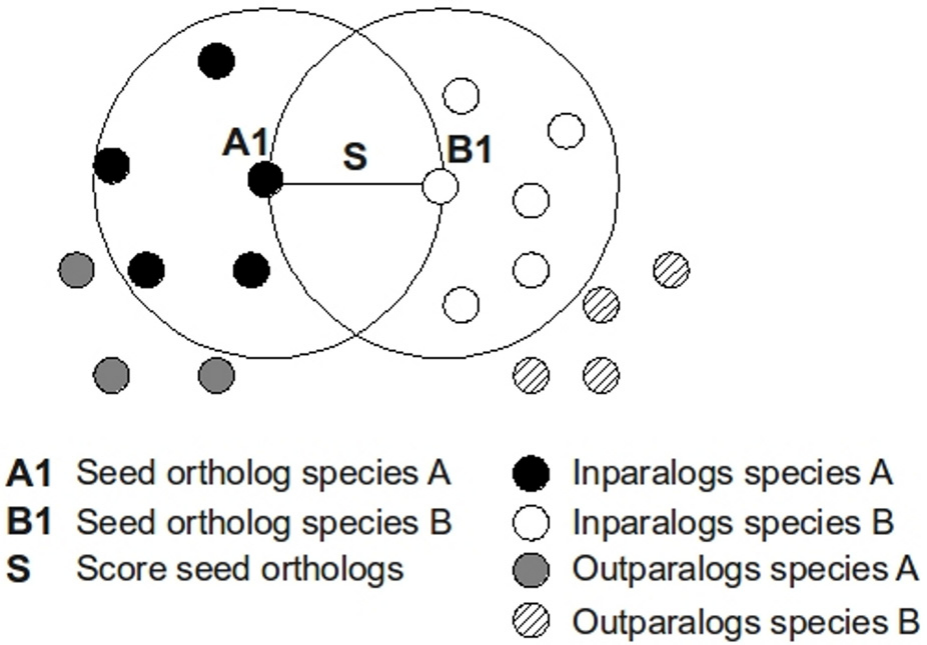
**Graphical representation of an InParanoid ortholog cluster with the outparalogs outside the cluster indicated**. The seed orthologs from the different species are denoted A1 and B1 and they are the bi-directional best Blast hits. Their similarity score (S) is shown. Inparalogs with score S or higher to the seed ortholog are inside the circle with radius S and hence, belonging to the cluster. Inparalogs are added to the cluster independently for each species. The sequences with a lower score than S are outside the cluster and classified as outparalogs. To generate the so-called extended cluster, for each inparalog in the cluster, the closest outparalog (non-ortholog or non-inparalog) from each species is added.

Several strategies have been employed for identifying orthologs, *e.g*. bidirectional best-hits (BBH) [3], InParanoid [4], OrthoMCL [5], KOG [6], Ensembl Compara [7], Homologene [8], EggNOG [9], and OMA [10]. These include both pairwise matching-based methods and tree-based methods, and they may also differ regarding whether they can assign orthology across two or several species. The performance of these strategies have been previously compared [11-13]. Although these comparative studies do not fully agree, it was found that InParanoid [4] is one of the most accurate pairwise ortholog assignment algorithms. Particularly when analyzing evolutionary relationships among eukaryotic genes it becomes very important to distinguish inparalogs from outparalogs, which methods based on simple two-way best matching fail to accomplish. Therefore, the InParanoid algorithm was designed to separate inparalogs, that are to be included in the cluster, from outparalogs, that are to be excluded, and also supplies a confidence score for the inparalogs in the cluster (figure 1). Moreover, the ortholog assignments are fully automatic and the algorithm is fast, thus enabling re-analysis of data upon new releases of genomes.

Ever since the discovery of introns their evolution has been studied. It has been shown that introns often maintain their positions over very long evolutionary timescales [14,15]. At these longer evolutionary distances, the sequence or length of the introns is never conserved. However, for very closely related species there might be selective pressure to maintain some intronic sequences due to presence of regulatory elements in the introns. Consequently, the conserved intron positions found between even distant species might be used to separate orthologs from other homologs. Indeed, this been done successfully in specific case studies of gene families, *i.e*. chemoreceptors [16,17], heat shock proteins [18], and homeobox genes [19]. Also, an algorithm called Exalign has been published where exon-intron gene structures are used to resolve phylogenetic relationships [20]. This method relies solely on exon lengths and phase, when available, to infer gene structural alignments. A drawback is that genes need to have at least four to five internal exons to produce high scoring alignments with a significant E-value, which limits its applicability.

Intron insertion is not a random process; they preferentially insert into or are fixed at so-called protosplice sites [21-24]. A study claimed that the majority of introns shared between distant species were the result of parallel gain into these sites [25]. These findings were later disputed and it was shown that protosplice sites are no more conserved during eukaryotic evolution than random sites [26]. In addition, simulation of intron insertion into protosplice sites with the observed protosplice sites frequencies and intron densities showed that parallel gain could account for only 5-10% of shared intron positions in distantly related species. Subsequently, this has been verified in other studies, where on average ~8% of shared intron positions in distantly related species were found to be due to parallel gain [27]. However, across the eukaryotic lineages, the distribution of parallel gain was highly heterogeneous with evolutionarily closer species showing virtually no shared introns due to parallel gain, whereas evolutionarily more distant species, such as human and plants, exhibited up to 20% parallel gain. A complicating factor when analyzing intron position conservation (IPC), is that different lineages exhibit very divergent rates and patterns of intron loss or gain [15,28,29]. It seems that intron loss is generally more prevalent than gain among orthologous genes [30-32], although there are studies showing that the opposite can sometimes be true [33].

The question still remains whether shared intron positions in different genes could be used on a global scale to aid the elucidation of evolutionary relationships, even between distant eukaryotic species. Therefore, in this study, we have analyzed the full genomes of seven eukaryotic species - human versus six other eukaryotes - to reveal if IPC can be used to distinguish orthologs from proteins that merely share amino acid similarity. More specifically, we examine if ortholog-ortholog (o-o) pairs have a higher IPC score compared to ortholog-closest non-ortholog (o-cno) pairs. In analogy, we also investigate whether inparalog-inparalog (i-i) pairs have a higher IPC score compared to inparalog-closest non-inparalog (i-cni) pairs. If this is the case, IPC could be used as a discriminatory variable when elucidating evolutionary relationships. Since sequences that are evolutionarily conserved tend to have a higher sequence identity compared to non-related sequences, we also examined the possible dependence between IPC and sequence identity. Finally, if IPC can be a predictor of orthology, it must agree at least to some extent with existing reliable orthology detection methods. Therefore, we analyzed the agreement between the InParanoid orthology score and the IPC score.

## Results

### A dataset of orthologs with intron positions

When analyzing intron conservation it is not feasible to take into account the actual intronic sequence or the intron length, since these features are generally not conserved due to lack of selective pressure. Despite this, an intron's position can be conserved over very long time spans. To analyze the conservation of intron positions, we generated a dataset of orthologs for seven eukaryotic species, where intron positions were indicated. We wanted to use human as the focal point, and then selected species on different evolutionary distance away from human. All of the selected species also have well-annotated genomes and a relatively high number of introns. The number of sequences were 26,815 for *Arabidopsis thaliana*; 20,140 for *Caenorhabditis elegans*; 14,039 for *Drosophila melanogaster; *21,322 for *Danio rerio*; 16,736 for *Gallus gallus*; 23,943 for *Homo sapiens*; and 24,166 for *Mus musculus*.

**Figure 2:**
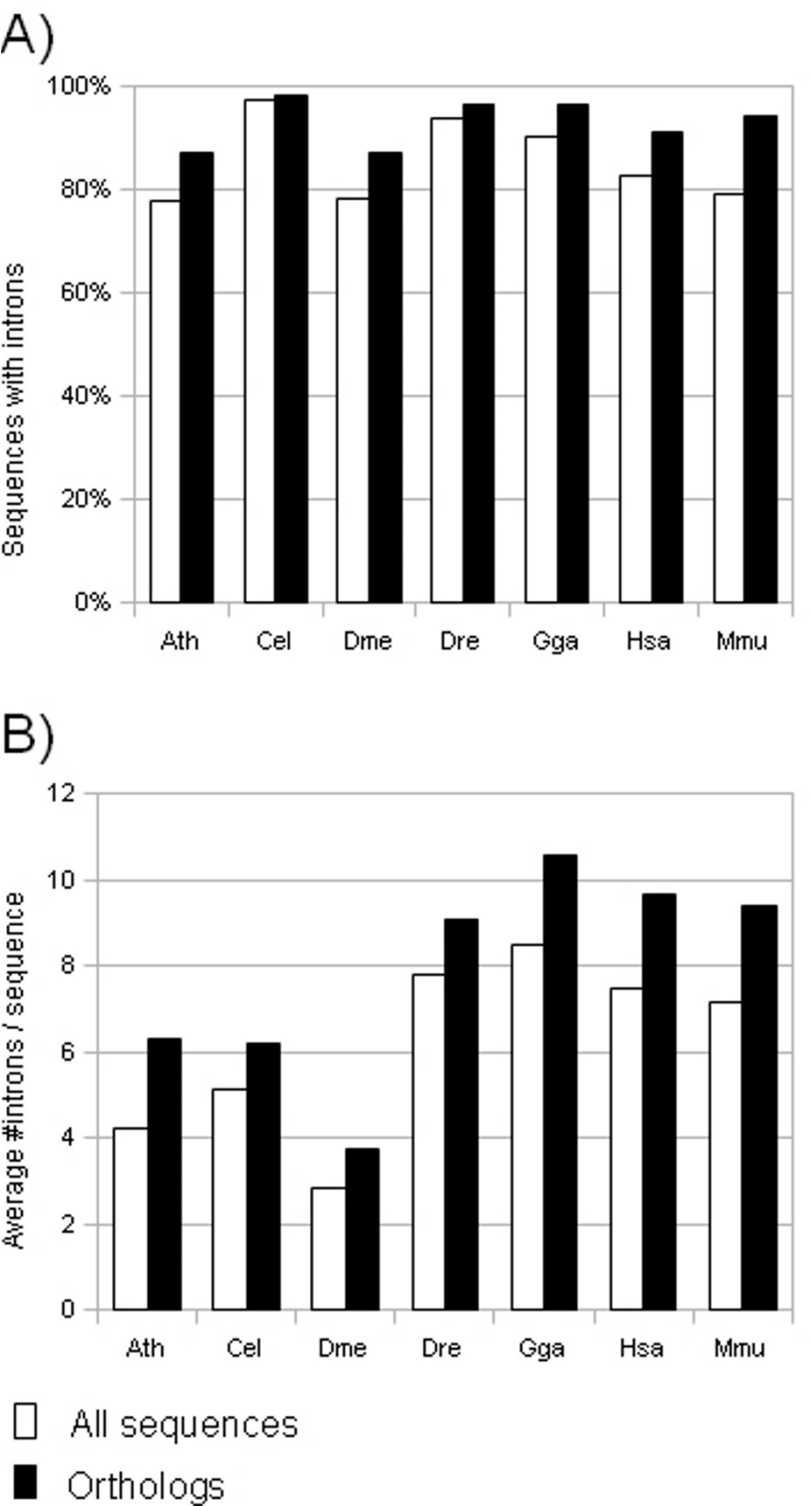
**Intron densities in the different genomes**. (A) Percentage of sequences harboring introns in the different genomes. (B) Average number of introns per sequence in the different genomes. All sequences means all protein coding genes in the genomes for each species. Orthologs means the subset of orthologs identified by the InParanoid algorithm for each species versus human. As a consequence, for human, orthologs refers to an average of the ortholog sets identified versus each of the other species.

We retrieved InParanoid ortholog clusters for human versus the six other species (table 1). Not surprisingly, the highest number of clusters were identified between human and mouse, of which a great majority are one-to-one ortholog clusters. Thereafter, human versus chicken and human versus zebrafish had the highest number of clusters, also with a great majority of one-to-one orthologs. Human versus Arabidopsis had the fewest clusters, although the number of orthologs was comparable to that of human versus Drosophila and human versus worm. This is mainly due to a greater number of duplications in Arabidopsis. When analyzing the distribution of introns in each species, we found that orthologs, regardless of species, are more likely to harbor introns compared to the genome as a whole (figure 2). Also, the average number of introns in orthologs is higher compared to all sequences in the studied species.

**Table 1:** InParanoid clusters and orthologs identified for the different species comparisons.

	#InParanoid ortholog clusters	#One-one Ortholog clusters^a^	#Multi Ortholog clusters^b^	#Orthologs Hsa	#Orthologs 2^nd ^species
**Hsa-Ath**	3144	1373	1771	6040	7939

**Hsa-Cel**	4507	2522	1985	8908	5737

**Hsa-Dme**	5302	3233	2069	8614	5983

**Hsa-Dre**	9899	8131	1768	11701	11957

**Hsa-Gga**	11081	10607	474	11796	11443

**Hsa-Mmu**	15309	14524	785	16274	16215

^a ^InParanoid clusters containing only seed orthologs.

^b ^InParanoid clusters containing seed orthologs and inparalogs.

**Figure 3:**
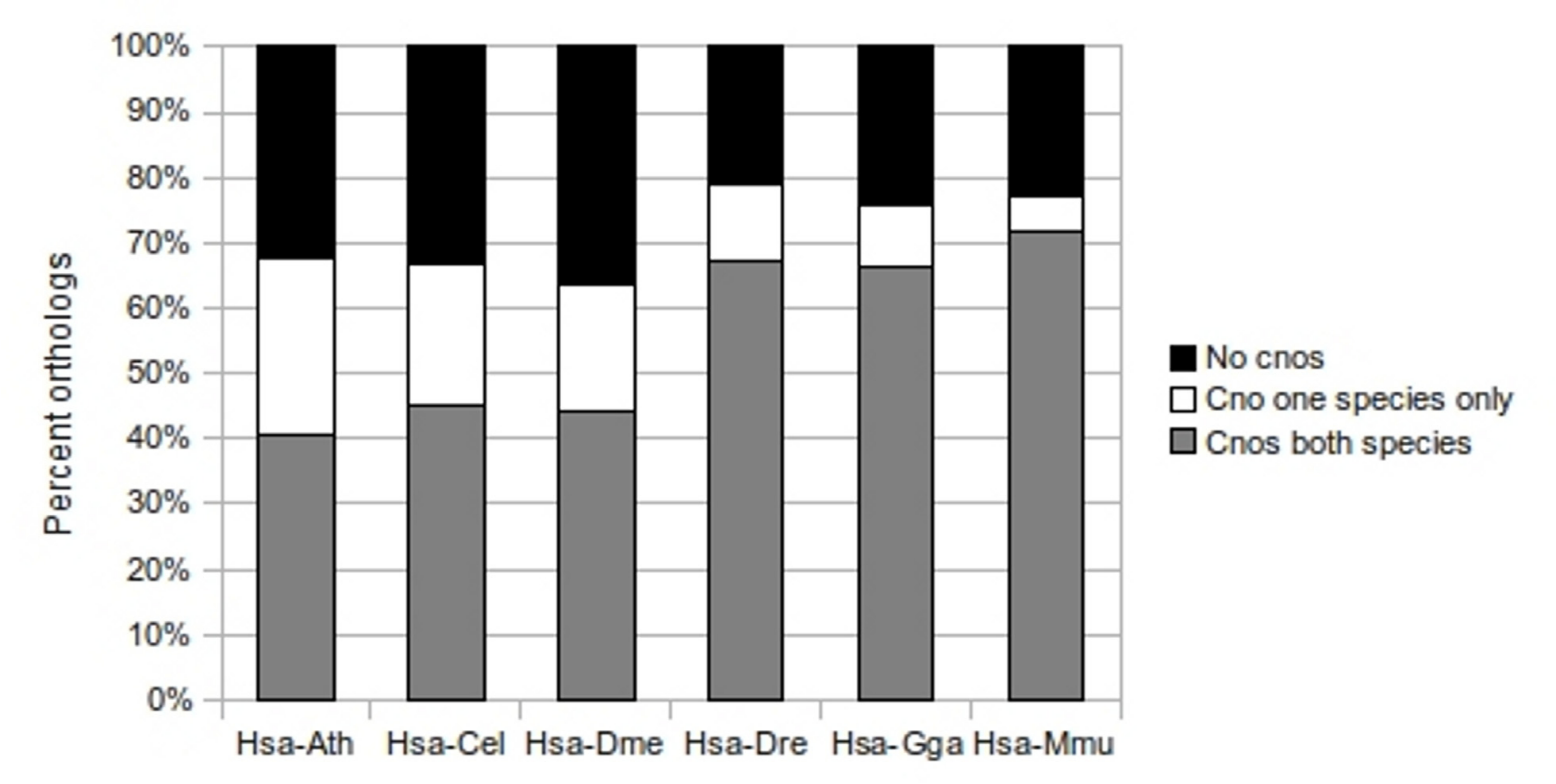
**Finding closest non-orthologs to add to the ortholog cluster**. Percent orthologs where a closest non-ortholog (cno) in either one or both species could be found, alternatively no cnos were found.

For this comparative study, the InParanoid clusters were extended by adding the closest non-ortholog (cno) and closest non-inparalog (cni) for each InParanoid cluster member. For human versus Arabidopsis, worm or Drosophila, a cno and cni could be found for roughly 43% of the inparalogs (figure 3), while for around 34% of the InParanoid cluster members neither a cno nor a cni could be found. For the remaining inparalogs, either a cno or a cni could be identified. For human versus chicken, zebrafish or mouse, there was a higher fraction of inparalogs where both a cno and a cni could be found (approximately 68%), whereas the percentage of inparalogs missing both cno and cni was lower (approximately 23%). This implies that for the vertebrate comparisons the ortholog cluster space is more densely populated and therefore makes it easier to find other homologs outside the ortholog group.

### Orthology versus intron position conservation

**Figure 4:**
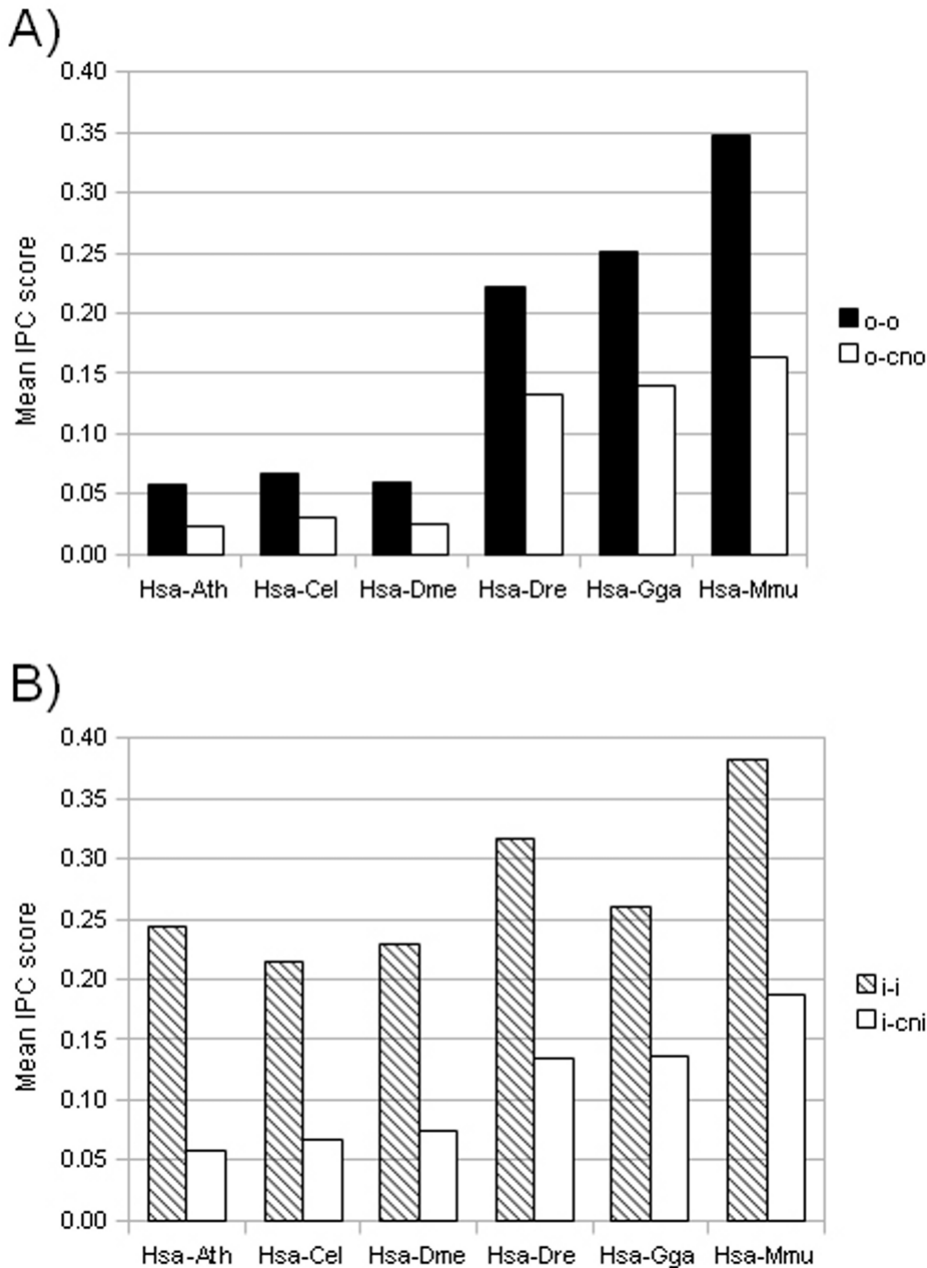
**Mean intron position conservation score for the different pair types and species comparisons (A) ortholog-ortholog (o-o) pairs versus ortholog-closest non-ortholog (o-cno) pairs, and (B) inparalog-inparalog (i-i) pairs versus inparalog-closest non-inparalog (i-cni) pairs**.

We wanted to examine the conservation of intron positions for orthologs compared to the closest non-orthologs. The idea being that if we can find a difference in intron position conservation (IPC) between these two groups, IPC score could be used as an additional feature for identifying orthologs. We scored the mean IPC for all pairs of the same type regardless of sequence identity. Across all species comparisons, the ortholog-ortholog (o-o) pairs had approximately twice as high mean IPC score compared to the ortholog-closest non-ortholog (o-cno) pairs (figure 4). Also, the the inparalog-inparalog (i-i) versus the inparalog-closest non-inparalog (i-cni) pairs showed a much higher mean IPC score for the i-i pairs. In fact, for all species comparisons except human versus mouse, the difference in mean IPC score was even greater than for the o-o versus o-cno pairs. We used the Mann-Whitney-Wilcoxon test to assess whether the IPC values for the o-o and o-cno pairs, and i-i and i-cni pairs, respectively, came from the same distribution or not. For all species comparisons, the IPC scores for the o-o pairs did not have the same distribution as the o-cno pairs (p-value < 0.05). The same was true for the i-i versus i-cni pairs. These results show that even when analyzed on a global scale, orthologs have a statistically significant higher IPC score than other homologs.

**Figure 5:**
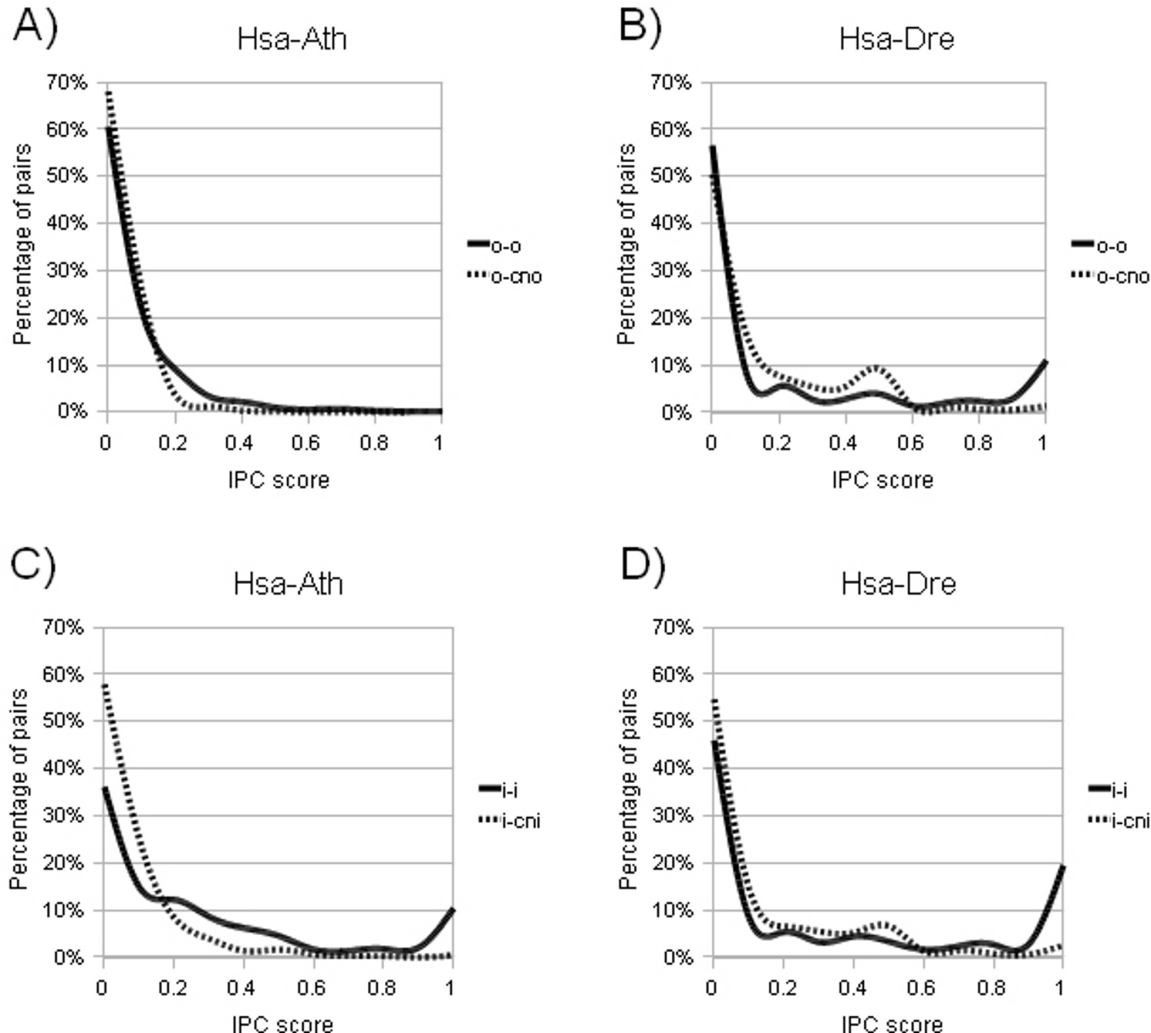
**Distribution of intron position conservation values for the different pair types**. (A) Hsa versus Ath, ortholog-ortholog (o-o) versus ortholog-closest non-ortholog (o-cno), (B) Hsa versus Dre, o-o versus o-cno, (C) Hsa versus Ath, inparalog-inparalog (i-i) versus inparalog-closest non-inparalog (i-cni), (D) Hsa versus Dre, i-i versus i-cni.

When examining the distribution of the IPC scores further, it becomes clear that it is very skewed (figure 5), [Additional file 1], [Additional file 2]. For human versus Arabidopsis, worm and Drosophila, a majority of pairs of all types, except i-i pairs, have no conserved intron positions. For human versus the vertebrates, approximately half of the o-o pairs as well as i-i pairs have an IPC score of zero. However, the number of o-cno and i-cni pairs with IPC score zero is almost always higher. These results suggest that even though not all orthologs have conserved intron positions, IPC score could still be used as a feature to separate orthologs from non-orthologs, because non-orthologs have an even lower degree of IPC. As exemplified by Arabidopsis and zebrafish in figure 5, the species closer to human (zebrafish, chicken and mouse) have a larger fraction of o-o pairs with high IPC score compared to the more distant species (Arabidopsis, worm and Drosophila). The same is true for the i-i pairs. This can be expected considering that a longer evolutionary time span increases the likelihood of introns being lost or gained, thereby decreasing the IPC score.

### Intron position conservation versus sequence identity

**Figure 6:**
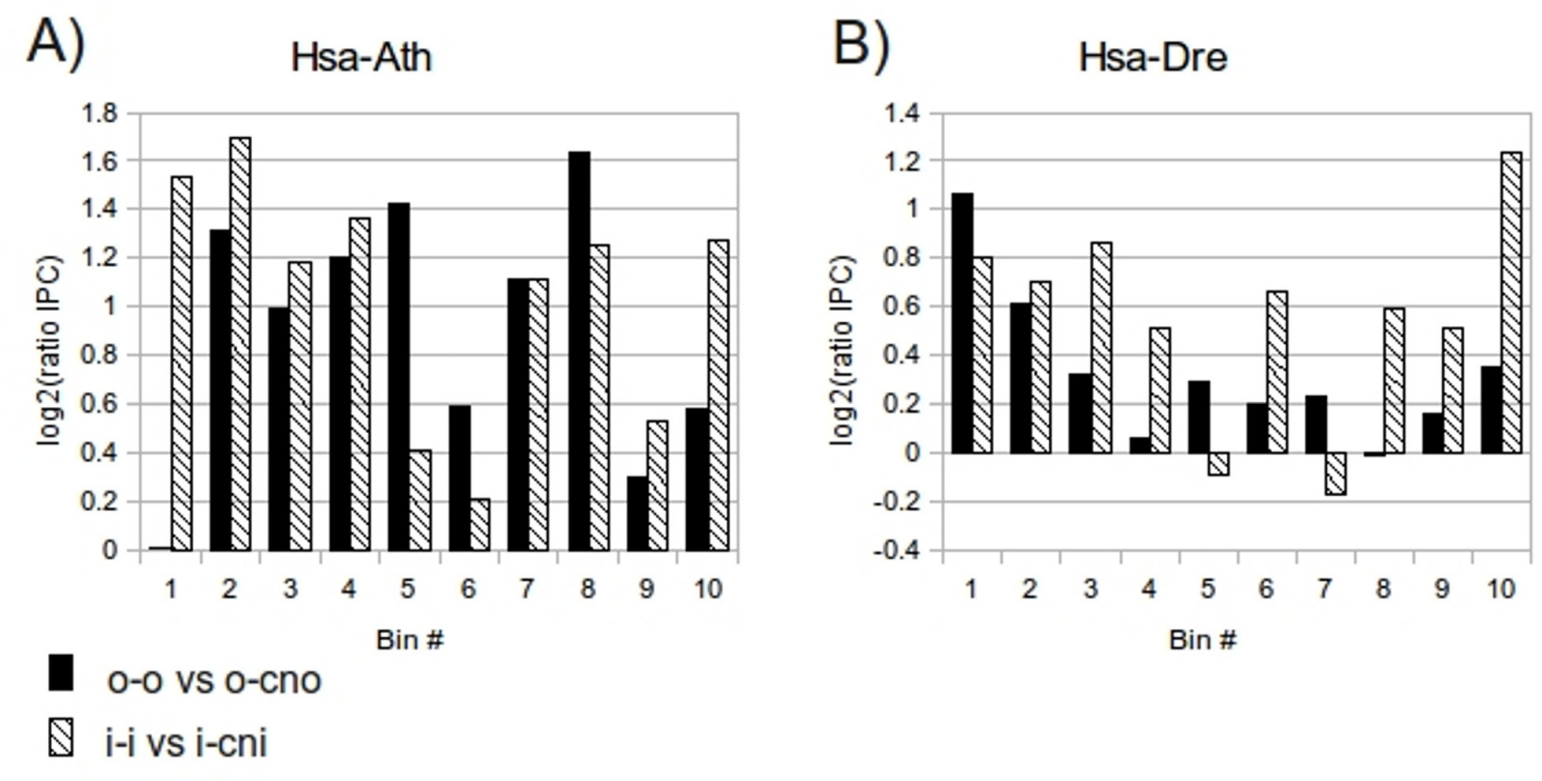
**Intron position conservation scores for pairs of the different types binned according to sequence identity**. Ortholog-ortholog (o-o) pairs versus ortholog-closest non-ortholog (o-cno) pairs, and inparalog-inparalog (i-i) pairs versus inparalog-closest non-inparalog (i-cni) pairs for (A) Hsa versus Ath, and (B) Hsa versus Dre.

Orthologs tend to have higher sequence similarity compared to non-orthologs. Could this account for their higher IPC score? In order to eliminate the effect of sequence similarity, we binned the pairs according to their sequence identity and then scored the IPC for each pair type and bin separately. The bin boundaries [Additional file 3], [Additional file 4] were chosen to distribute the different pair types as equally as possible among the bins. In a great majority of the bins, the o-o and i-i pairs have a higher degree of intron position conservation compared to their non-ortholog counterparts even when normalizing for sequence identity (figure 6), [Additional file 5]. This is true for all species comparisons analyzed. This shows that orthologs, even when adjusting for their higher sequence identity, have more conserved intron positions compared to non-orthologs. Several different number of bins were tested, however, the results were essentially the same.

To further investigate the possible dependence between sequence identity and IPC score, we calculated the Spearman correlation coefficient between them for all pair types and species comparisons. The correlation coefficient between IPC score and sequence identity was generally low (<0.3) (table 2), although in most cases statistically significant at the 5% level. We note that o-cno pairs always had a higher correlation than o-o pairs, suggesting that the IPC scores of o-cno pairs are more explainable by sequence identity. The results from both the binning of pairs according to their sequence identity and the Spearman correlation analysis, indicate that the higher IPC score of orthologs compared to non-orthologs cannot be explained simply by the higher sequence identity of the former.

**Table 2:** Correlation between intron position conservation score and sequence identity.

	*o-o^a^*	*o-cno^b^*	*i-i^c^*	*i-cni^d^*
**Hsa-Ath**	0.07*	0.12*	0.05*	0.22*

**Hsa-Cel**	0.01	0.15*	-0.01	0.16*

**Hsa-Dme**	0.07*	0.15*	0.04	0.16*

**Hsa-Dre**	0.11*	0.28*	0.21*	0.17*

**Hsa-Gga**	0.09*	0.25*	0.09	0.06

**Hsa-Mmu**	0.13*	0.21*	0.17*	0.11*

Spearman correlation coefficient for intron position conservation score versus sequence identity for the different species comparisons and pair types.

^a ^ortholog-ortholog pair

^b ^ortholog-closest non-ortholog pair

^c ^inparalog-inparalog pair

^d ^inparalog-closest non-inparalog pair

*P-value < 0.05

### Intron position conservation score versus InParanoid ortholog score

The InParanoid algorithm [4] can find non-overlapping clusters of orthologs and inparalogs across two species. The algorithm first finds the bi-directionally best Blast hits between the two genomes, the so-called **seed **orthologs. Around these seed orthologs, inparalogs from each species are clustered separately (figure 1). Sequences in the same species that are more similar to the seed ortholog than to any sequence in the other species will be classified as an inparalog and added to the cluster. The inparalogs are ranked by a confidence score that is calculated for each inparalog, reflecting its similarity to the seed ortholog. In a previous study, the correlation between protein-protein interaction (PPI) and the InParanoid inparalog ranking was assessed [34]. They analyzed 121 cases where the ranking was called "ambiguous", meaning that the ortholog cluster is not a one-to-one cluster, but a "multi-cluster". For approximately half of these, the PPI network suggested a different ranking than that proposed by InParanoid. In analogy, we wanted to examine how well InParanoid's ranking of inparalogs in an ortholog group agrees with their IPC score to the seed ortholog in the other species. Furthermore, if IPC is to be used when inferring evolutionary relationships, it must agree at least to some extent with existing reliable orthology detection methods.

The analyzed clusters were split into "one-to-one", meaning those that only contain seed orthologs, and "multi" clusters, meaning those that also contain inparalogs. For the human-vertebrate comparisons, the great majority of the analyzed clusters are one-to-one clusters, whereas for the others there is a large fraction of multi-clusters (table 1). First, we analyzed the multi-cluster seed ortholog assignments agreement with IPC score, i.e. does the seed ortholog pair (or possible spliceforms thereof) have the highest IPC score of the possible ortholog pairs in the cluster? We found that for the great majority of multi-clusters, IPC score supports the seed ortholog assignments made by the InParanoid algorithm (>70% for human versus Arabidopsis, worm and Drosophila, and >80% for human versus vertebrate comparisons).

To further assess the correlation between IPC score and multi-cluster seed ortholog assignments, we considered the bootstrap value that InParanoid assigns each seed ortholog. This indicates the confidence in the "seed" ranking of the inparalog as the fraction of intracluster bootstrap runs that placed it as the best match. The multi-clusters were split according to their seed ortholog bootstrap support, and the agreement with IPC score was investigated. For multi-clusters where the seed orthologs have a bootstrap support of at least 90% there is a higher agreement with IPC, meaning that the seed ortholog pair also have the highest IPC score, compared to when the bootstrap is below 90% (table 3). There is thus a correlation between IPC score and InParanoid seed ortholog assignments, meaning that a high IPC score generally implies a highly confident orthology relationship. On the other hand, as in the study by [34], we found a substantial number of ortholog groups, ranging from 82 in human versus chicken to 504 in human versus Arabidopsis, where the inparalog ranking of external evidence did not agree with InParanoid. This highlights the importance of considering all inparalogs when using orthology for annotation transfer between species.

**Table 3:** Agreement between InParanoid seed ortholog assignment and intron position conservation score.

	Seed ortholog bootstrap >= 90%	Seed ortholog bootstrap <90%
**Hsa-Ath**	73%	67%

**Hsa-Cel**	76%	70%

**Hsa-Dme**	81%	78%

**Hsa-Dre**	81%	72%

**Hsa-Gga**	87%	63%

**Hsa-Mmu**	88%	70%

Percentage of InParanoid clusters where the assigned seed orthologs also have the highest intron position conservation score of the possible ortholog pairs.

### Function term enrichment analysis and IPC-orthology disagreement

Is conservation of intron position, or the lack thereof, associated with some specific classes of proteins, such as those belonging to certain pathways or cellular roles? To answer this question, we evaluated whether or not the distribution of Gene Ontology [35] terms was the same for proteins where IPC and evolutionary distance were in agreement and proteins where they disagreed. These measures are considered to agree for InParanoid clusters where the seed ortholog pair has the highest IPC of all the ortholog pairs in the cluster. We say that such clusters are consistent. Because of this, agreement status is only well-defined for clusters where subsequent gene duplication has occurred, so called multi-clusters. Two multi-clusters of olfactory receptors, one between human and worm and one between human and zebrafish, containing more than 200 genes each, were rejected on grounds of size as potential artifacts in the orthology analysis. If included in the functional enrichment analysis, the associated functions and 7tm_1 domains would appear significantly enriched.

As the degree and quality of Gene Ontology annotation varies significantly between species, only the human protein annotations were used in the analysis. Of 11,732 human genes present in multi-clusters in at least one of the species comparisons, 6,725 were found only in consistent clusters, whereas 2,074 were found only in inconsistent clusters. Only these genes (about 75% of all multi-cluster proteins) were used for the analysis, as genes found in both consistent and inconsistent clusters in different species comparisons could not unambiguously be associated with either cluster category. 30% of these genes were present in multi-clusters in more than one species comparison.

As can be seen in [Additional file 6], some trends are visible. Since many Gene Ontology terms are either associated by parent-child relationships or associated in practice because they generally co-occur, it is possible to summarize the set of enriched functional terms into some broad categories. A Gene Ontology term in this table is considered to derive from another term if exclusion of proteins annotated with the latter term from the analysis would make the former term no longer significantly enriched. Following a procedure described in greater detail below, we clustered the enriched terms based on such associations, and selected the most highly connected terms in the resulting graph as representatives for the enriched term set as a whole. Most (43 of 50 terms) of the enriched terms turned out to be associated with GO:0016020, "membrane", while most of the remainder (5 of 50 terms) were associated with GO:0016773, "phosphotransferase activity, alcohol group as acceptor". In addition, the terms GO:0005581, "collagen", and GO:0001533, "cornified envelope", were individually enriched. Terms associated with GO:0016020 included examples such as GO:0042611, "MHC protein complex", GO:0022857, "transmembrane receptor protein kinase activity", GO:0019882, "antigen processing and presentation", and GO:0007155, "cell adhesion", encoding functions important for multicellularity. The set of terms associated with GO:0016773 include examples such as GO:0004713, "protein tyrosine kinase activity", which is also a hallmark of complex multicellular organisms.

Conversely, the representative functions for the set of depleted Gene Ontology terms were GO:0043231, "intracellular membrane-bounded organelle" (63 of 68 terms), GO:0003735, "structural constituent of ribosome" (4 of 68 terms) and GO:0009057, "macromolecule catabolic process" (1 of 68 terms). These fundamental and ancient housekeeping functions, important also for single-cell organisms, are thus more often found in the consistent multi-clusters. One possible interpretation of these results is that the creation of multicellularity gave rise to disagreement between intron position and sequence conservation, perhaps due to rapid adaptation into entirely new functional niches needed to maintain a multicellular organism. Possibly this happened by exon shuffling, a mechanism that would make this process faster and would frequently result in altered intronic structure.

### Protein family enrichment analysis and IPC-orthology disagreement

We also made Pfam [36] domain assignments for the same sets of genes as above, and generalized to higher-level clan assignments where possible. The set of domains in each protein was considered, and an enrichment/depletion analysis was performed using the same methods and tools as for the functional class enrichment analysis. There is less of a clear trend visible at the domain level [Additional file 7] compared to the functional enrichment analysis; however, the results are broadly compatible with the general trends we observed: enrichment of MHC-associated domains, protein kinase domains, ion channels and cell adhesion-related domains such as collagen and cadherin.

### Protein properties analysis and IPC-orthology disagreement

We also analyzed whether proteins in the above subsets differed with regards to their length, their number of domains, or their number of introns. The human proteins found only in inconsistent multi-clusters were on average ~25% longer, had ~37% more Pfam-A domains, and had ~44% more introns than the human proteins found only in consistent clusters. These differences, while modest in strength, were highly significant (p < 2.2e-16). Although it is possible that the number of introns per protein may affect the *a priori *probability of a cluster becoming consistent or inconsistent, it is not clear whether this probability would increase or decrease. In any case, as the distributions of all three properties are highly overlapping between the two subsets (data not shown), it seems unlikely that this difference would be a major factor behind determining whether a cluster exhibits IPC-orthology disagreement or not. As the proteins found only in inconsistent clusters are enriched for functions associated with multicellularity, it is not unexpected that they should also be longer and contain more domains and introns.

## Discussion

We have presented a global study which show that ortholog-ortholog (o-o) and inparalog-inparalog (i-i) pairs in seven different eukaryotes have a higher degree of intron position conservation (IPC) compared to their respective ortholog-closest non-ortholog (o-cno) and inparalog-closest non-inparalog (i-cni) pairs. We have also shown that this difference in IPC cannot be explained merely by the fact that o-o and i-i pairs have a higher sequence identity. There is a weak correlation between sequence identity and IPC score, which is to be expected considering that it has been shown that introns preferentially insert into or are fixed at so-called protosplice sites [21-24]. Due to the presence of these protosplice sites, it has been suggested that the conservation of intron positions observed in orthologs is simply due to independent insertion of introns in the same sites (parallel gain) [25]. However, it has been shown that such parallel gain can only account for on average ~8% of the conserved intron positions [27]. For certain lineages the number can be higher, but the great majority of shared intron positions is due to conservation of ancestral introns.

We show that all species comparisons have approximately two-fold higher mean IPC score for the o-o pairs compared to o-cno pairs; however, there is a large difference in mean IPC value for the different species comparisons. Human-mouse orthologs had approximately six times higher mean IPC compared to more evolutionarily distant species (Arabidopsis, worm and Drosophila). Human-zebrafish and human-chicken orthologs had four times higher mean IPC score than the more evolutionarily distant species. This is simply reflecting the difference in evolutionary distance between human and the various species in the analysis. Orthologs in distantly related species have been separated a longer time compared to closely related species, and as a consequence they are more likely to have diverged in sequence and therefore share less intron positions. On the other hand, recently duplicated sequences such as inparalogs, are more likely to have conserved intron positions due to the shorter evolutionary time since the duplication event. Indeed, for the i-i pairs across all species comparisons, we find a much higher IPC score compared to the o-o pairs. Notably, the difference in IPC score between the i-i and i-cni pairs is much lower for human versus the evolutionarily closer species (zebrafish, chicken and mouse) compared to human versus the others. This is due to the fact that cnis are sequences which predate the species split, and therefore cnis in human versus the evolutionarily closer species have had less time to diverge compared to the cnis in the more distant species. This can also be seen in the distribution of IPC values (figure 5), where the evolutionarily closer species have more o-cno and i-cni pairs than o-o and i-i pairs, respectively, in the middle range of IPC values, whereas that is not the case for the more distant species. Also, there is a slight trend that the further away on the evolutionary timescale compared to human, the lower the mean IPC score for the i-i pairs. One can speculate that this happens because inparalogs in the more distant evolutionary species have arisen earlier during evolution compared to inparalogs in the evolutionarily closer species.

Even though our results show a higher IPC score for o-o and i-i pairs, there is still a large fraction of both of these pair types that have no conservation of intron positions. This could be due to a number of reasons; however, they mainly fall into two categories: limitations in the dataset and the nature of intron evolution. When it comes to limitations in the dataset, they are inevitably numerous since assigning intron positions in the genomic sequence is not a trivial task. Therefore, introns might incorrectly be missing, present, or misplaced. With time the datasets will become more complete and allow for a more accurate understanding of the conservation of intron positions. Biological reasons for the lack of IPC includes great differences in the intron loss/gain patterns or in rates for different lineages [15,28,29]. Also, "intron sliding" has been proposed to happen during evolution [14], although this has been shown to be a rare phenomenon [37,38]. In our study, we considered an intron position as conserved if found within the same codon, meaning that at most the intron is allowed to slide 2 nucleotides. We also tried allowing a greater slide, however, this resulted in worse separation between the ortholog and non-ortholog pairs, implying that non conserved intron positions were scored as being conserved.

We find that the inparalog ranking made by InParanoid is largely corroborated by the IPC score. Especially, in a great majority of the multi-clusters, IPC score support the seed ortholog designation made by InParanoid. We also find that clusters where IPC score suggests a different seed ortholog pair tend to have a lower bootstrap support, meaning that the InParanoid inparalog ranking is slightly less certain. The fact that IPC to a great extent agrees with the InParanoid inparalog ranking, shows that IPC can indeed be used as a additional discriminating factor for determining evolutionary relationships among genes. Among those clusters where IPC score is inconsistent with the InParanoid inparalog ranking, we find significant enrichment of certain protein functions. However, due to the low fraction of genes present in multi-clusters in more than one species comparison, which follows from our selection of species, there is a risk that this result is influenced by lineage-specific conditions, and so caution should be applied when interpreting its global biological significance. The observed enrichment of certain functional classes might reflect mutational mechanisms and selective pressures operating before and after the origin of complex, multicellular organisms. Further studies correlating functions to different gene family evolutionary ages or to lineages should be undertaken before general conclusions can be drawn.

## Conclusions

In summary, we have shown that evolutionarily related genes have more conserved intron positions compared to merely similar sequences. However, our observations disclose that a low IPC score does not necessarily mean that two genes are not orthologs. On the other hand, if two genes have a high IPC score they are much more likely to be orthologs. Hence, a high IPC score can be used to further discriminate orthologs from non-orthologs. A possible application would be to take intron positions into account when performing sequence alignments by giving a higher score if intron positions are conserved. Consequently, current orthology detection methods could benefit from incorporating information on intron positions.

## Methods

### Datasets

The EMBL flat files for the full genomes of *Caenorhabditis elegans*, *Drosophila melanogaster*, *Danio rerio*, *Gallus gallus*, *Homo sapiens*, and *Mus musculus *were downloaded from the Ensembl database [39]. The GenBank flat files for the full genome of *Arabidopsis thaliana *was downloaded from NCBI [40]. All files were downloaded on November 15^th ^2007, except for zebrafish and chicken which were downloaded on 7^th ^February 2008. The files were processed to extract all genes and their corresponding protein sequences. The genomic positions of introns were taken from the "join" and "join(complement)" locations given in the CDS feature key in the feature table of the flat files. The qualifiers for protein_id and translation were also extracted. For the EMBL flat files, the qualifier for gene was selected be able to filter for spliceforms. For the GenBank flat files, the qualifier db_xref:GeneID was retrieved for the same purpose. The positions of introns were mapped to the amino acid sequence of each protein, in such a way that if the intron was just preceding or interrupting a codon, the equivalent amino acid was set to a lower case letter in the protein sequence. Potential redundancy (identical gene copies) was removed and subsequently the sequences were filtered to keep only the longest spliceform.

### Computing ortholog clusters

The resulting sequence files were Blasted all-against-all (human versus each of the other species) using blastp [41] and then the InParanoid algorithm [4] version 3.1 was used to identify ortholog clusters. The sequence overlap cutoff was 0.5 and segment coverage cutoff was 0.25.

Each InParanoid ortholog cluster was further extended by adding the closest non-ortholog (cno) and closest non-inparalog (cni) for each cluster member (figure 1). By this, we mean the best match from each species in the Blast results that were not already members of the cluster. These sequences also had to meet the same sequence overlap cutoff and segment coverage cutoff as the InParanoid cluster members. Clusters for mitochondrial genes were excluded since all such sequences are intronless. Finally, for each InParanoid cluster member, any shorter spliceform was added back to the cluster prior to scoring the conservation of intron positions in these so-called extended clusters.

### Scoring intron position conservation

All protein sequences in a so-called extended cluster were multiply aligned using Kalign 2.0 [42]. For a few of the extended clusters it was impossible to generate a sequence alignment and therefore they were excluded. This occurred when the extended cluster contained sequences of highly different lengths (several thousand amino acids), making it impossible for the algorithm to construct an alignment. The conservation of intron positions was scored pairwise for all orthologs and inparalogs and their corresponding closest non-orthologs and closest non-inparalogs. Hence, there are four different types of pairs that are analyzed, namely ortholog-ortholog (o-o), inparalog-inparalog (i-i), ortholog-closest non-ortholog (o-cno), and finally, inparalog-closest non-inparalog (i-cni) pairs. Intron positions were considered as conserved if present within the same codon, meaning that the intron position can slide a maximum of 2 nucleotides and still be scored as conserved. We also extracted the sequence identity given by Kalign. If an ortholog (inparalog) had several spliceforms, the o-o (i-i) pair with the highest intron position conservation (IPC) score was chosen. For each o-o (i-i) pair, there can be two o-cno (i-cni) pairs. If this was the case, the mean IPC score and sequence identity of the two pairs was calculated. Finally, the mean IPC score and sequence identity for each of the four possible pair types in an extended cluster was calculated separately. The Mann-Whitney-Wilcoxon test was used to assess whether the different pair types came from the same distribution or not.

The intron position conservation for a pair of sequences was calculated in the following way:

where IPC is intron position conservation, introns_shared _is the number of introns that share the same position in the two sequences compared allowing for a slide within the same codon, introns_seqA _is the number of introns in sequence A, and introns_seqB _is the number of introns in sequence B.

To examine whether sequence similarity alone could explain a higher IPC score, the pairs were binned on sequence identity. To achieve a sufficient number of pairs of each type in all bins, the o-o and o-cno pairs, and i-i and i-cni pairs, respectively, were binned separately in the same way, described as follows. First, the two different pair types were binned separately into ten bins with the same number of pairs in each bin. From the obtained sequence identity bin boundaries, new combined bin boundaries were calculated by taking the average of the individual bin boundaries for the two pair types.

Subsequently, the data was redistributed according to these new bin boundaries.

The log_2_(ratio IPC) was calculated for each bin in the following way:

where mean IPC_o-o (i-i) _is the mean IPC score for all the o-o (i-i) pairs in that bin and mean IPC_o-cno (i-cni) _is the mean IPC score for all o-cno (i-cni) pairs in the same bin. Consequently, log_2_(ratio IPC) = 0 means that the two pair types compared have the same mean IPC score. To further assess the possible correlation between IPC score and sequence identity, the Spearman correlation coefficient for each pair type and species comparison was also calculated using R.

### Functional class enrichment analysis

Gene Ontology annotations were downloaded on March 9 2009 from ENSEMBL BioMart [43]. The set of human genes found only in inconsistent multi-clusters (*i.e*. where orthology assignment and IPC did not agree) was contrasted against a background consisting of this set together with the set of genes found only in consistent multi-clusters. Two multi-clusters, one between human and worm (705 genes) and one between human and fish (398 genes), were removed because of their large size, which we interpreted as an indication of potentially incorrect orthology assignments. The genes in both clusters encode olfactory receptors, most of which have no introns. All Gene Ontology terms assigned to any of the genes in the set were tested for enrichment or depletion among the genes belonging only to inconsistent clusters. We calculated the probability of these observations under the null hypothesis of no enrichment or depletion using a hypergeometric distribution [44]. The procedure was implemented in-house as a simple Perl script. To avoid drawing erroneous conclusions from multiple testings, the False Discovery Rate (FDR) was controlled at 1% using the procedure from [45], meaning the fraction of false positives among the terms inferred enriched or depleted is expected to be below 1%.

Gene Ontology terms often have complex interdependencies, e.g. terms that are ancestors or children of each other, or terms that always or very often co-occur. These interdependencies must be taken into account when analyzing a set of enriched or depleted terms. We selected representative terms for major trends within the lists of significantly enriched or depleted terms as follows. For each term A in the list, enrichment/depletion was recomputed for the subset of the proteins resulting from exclusion of all proteins annotated with A from the dataset. Any term B which is significantly enriched/depleted using the full set of proteins, but no longer when A-annotated proteins were excluded, was considered to be associated with A. These associations form the links of a network of Gene Ontology terms. Within this network, we selected as the first representative term C1 which had the most links to other terms in the network. C1 and all its direct neighbors were then removed, and the remaining most highly connected term C2 was selected, and the procedure repeated, until all terms belonged to a term subset with an associated most highly connected representative.

### Protein family enrichment analysis

We also made Pfam-A [36] domain assignments for the same sets of genes as above. The set of domains in each protein was considered, and an enrichment/depletion analysis was performed using the same methods and tools as the GO term analysis described in the previous section. Domains were replaced in this analysis with Pfam clans where available, to reflect wider categories of likely homologous domains.

### Protein length analysis

We compared the two subsets of proteins tested for functional enrichment above with respect to protein length in amino acids, number of introns, and number of Pfam-A domains, where consecutive stretches of the same Repeat/Motif-type Pfam-A domain were collapsed into a single pseudo-domain, as repeat differences of this type are extremely variable. For each length measure, the distribution of lengths across the two subsets were compared, under a null hypothesis of the distributions being the same, using the Mann-Whitney U test/Wilcoxon rank sum test [46], as implemented in the R [47] software.

## List of Abbreviations

cni: closest non-inparalog; cno: closest non-ortholog; i-cni: inparalog-closest non-inparalog; i-i: inparalog-inparalog; IPC: intron position conservation; o-cno: ortholog-closest non-ortholog; o-o: ortholog-ortholog.

## Authors' contributions

AH and KF carried out the analysis and wrote the manuscript. ES conceived of the study, participated in the analysis and helped to draft the manuscript. All authors read and approved the final manuscript.

## Supplementary Material

Additional file 1 **Distribution of intron position conservation values**. Distribution of intron position conservation values for the different pair types in human versus worm and human versus Drosophila. (A) Hsa versus Cel, ortholog-ortholog (o-o) versus ortholog-closest non-ortholog (o-cno), (B) Hsa versus Dme, o-o versus o-cno, (C) Hsa versus Cel, inparalog-inparalog (i-i) versus inparalog-closest non-inparalog (i-cni), (D) Hsa versus Dme, i-i versus i-cni. Click here for file

Additional file 2 **Distribution of intron position conservation values**. Distribution of intron position conservation values for the different pair types in human versus chicken and human versus mouse. (A) Hsa versus Gga, ortholog-ortholog (o-o) versus ortholog-closest non-ortholog (o-cno), (B) Hsa versus Mmu, o-o versus o-cno, (C) Hsa versus Gga, inparalog-inparalog (i-i) versus inparalog-closest non-inparalog (i-cni), (D) Hsa versus Mmu, i-i versus i-cni. Click here for file

Additional file 3 **Bin boundaries and number of pairs in the different bins**. Sequence identity bin boundaries and number of ortholog-ortholog (o-o) and ortholog-closest non-ortholog (o-cno) pairs in the different bins. The structure of data in each bin is given above the table. Click here for file

Additional file 4 **Bin boundaries and number of pairs in the different bins**. Sequence identity bin boundaries and number of inparalog-inparalog (i-i) and inparalog-closest non-inparalog (i-cni) pairs in the different bins. The structure of data in each bin is given above the table. Click here for file

Additional file 5 **Intron position conservation versus sequence identity**. Intron position conservation scores for pairs of the different types binned according to their sequence identity shown for human versus four other species. Ortholog-ortholog (o-o) pairs versus ortholog-closest non-ortholog (o-cno) pairs, and inparalog-inparalog (i-i) pairs versus inparalog-closest non-inparalog (i-cni) pairs for (A) Hsa versus Cel, (B) Hsa versus Dme, (C) Hsa versus Gga, and (D) Hsa versus Mmu. Click here for file

Additional file 6 **Enrichment or depletion of GO terms in the inconsistent clusters**. GO terms significantly enriched or depleted in the inconsistent clusters, *i.e*. clusters where the IPC score and the InParanoid inparalog ranking did not agree. Enriched/depleted GO terms are divided into uniquely colored groups of associated terms, each with a most highly connected representative listed at the top. Click here for file

Additional file 7 **Enrichment or depletion of Pfam domains in the inconsistent clusters**. Pfam domains significantly enriched or depleted in the inconsistent clusters, *i.e*. clusters where the IPC score and the InParanoid inparalog ranking did not agree. Clans are used in place of domains where available. Click here for file
