# Supplementary material for: Orthology confers intron position conservation
Source: BMC Genomics. 2010 Jul 2;11:412. doi: 10.1186/1471-2164-11-412 (PMC2996940; doi:10.1186/1471-2164-11-412)
Supplement: Additional file 1 — Distribution of intron position conservation values. Distribution of intron position conservation values for the different pair types in human versus worm and human versus Drosophila. (A) Hsa versus Cel, ortholog-ortholog (o-o) versus ortholog-closest non-ortholog (o-cno), (B) Hsa versus Dme, o-o versus o-cno, (C) Hsa versus Cel, inparalog-inparalog (i-i) versus inparalog-closest non-inparalog (i-cni), (D) Hsa versus Dme, i-i versus i-cni. [file 1471-2164-11-412-S1.PDF]

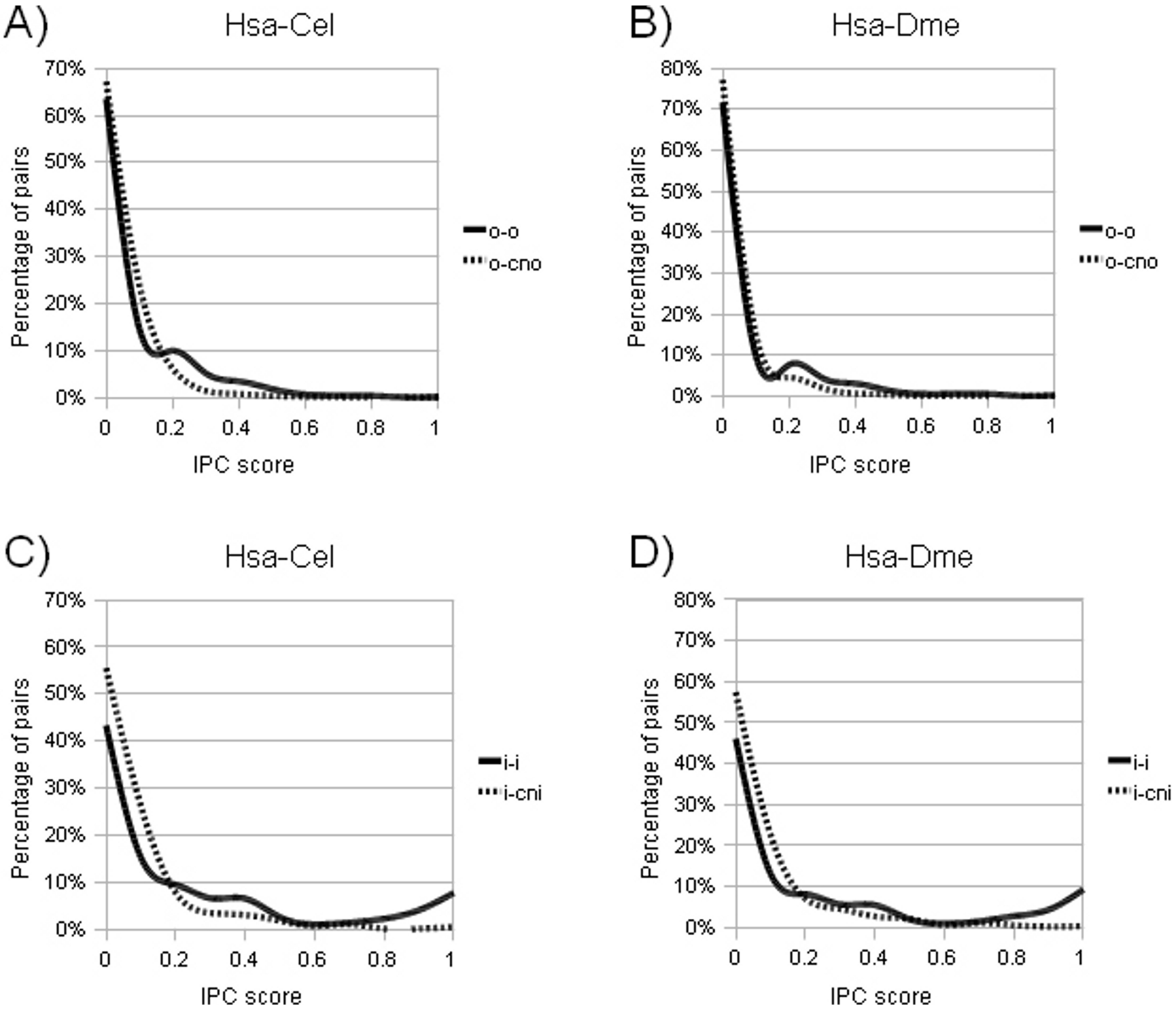

Figure S1. Distribution of intron position conservation values for the different pair types in human versus worm and human versus Drosophila. (A) Hsa versus Cel, ortholog-ortholog (o-o) versus ortholog-closest non-ortholog (o-cno), (B) Hsa versus Dme, o-o versus o-cno, (C) Hsa versus Cel, inparalog-inparalog (i-i) versus inparalog-closest non-inparalog (i-cni), (D) Hsa versus Dme, i-i versus i-cni.
