# Supplementary material for: Orthology confers intron position conservation
Source: BMC Genomics. 2010 Jul 2;11:412. doi: 10.1186/1471-2164-11-412 (PMC2996940; doi:10.1186/1471-2164-11-412)
Supplement: Additional file 3 — Bin boundaries and number of pairs in the different bins. Sequence identity bin boundaries and number of ortholog-ortholog (o-o) and ortholog-closest non-ortholog (o-cno) pairs in the different bins. The structure of data in each bin is given above the table. [file 1471-2164-11-412-S3.PDF]

Table S1. Sequence identity bin boundaries and number of ortholog-ortholog (o-o) and ortholog-closest non-ortholog (o-cno) pairs in the different bins. The structure of data in each bin is given above the table.

Bin #	
% sequence identity bin boundaries	
#o-o[a] pairs	#o-cno[b] pairs

	Bin #1		Bin #2		Bin #3		Bin #4		Bin #5		Bin #6		Bin #7		Bin #8		Bin #9		Bin #10	
**Hsa-Ath**	0-19.39		19.40-22.97		22.98-25.73		25.74-28.23		28.24-30.98		30.99-33.74		33.75-37.16		37.17-41.39		41.40-48.41		48.42-100	
	195	*324*	*192*	*276*	*237*	*245*	*242*	*225*	*298*	*202*	*251*	*161*	*322*	*143*	*366*	*106*	*430*	*86*	*611*	*81*
**Hsa-Cel**	0-20.35		20.36-23.81		23.82-26.82		26.83-29.46		29.47-32.09		32.10-35.14		35.15-38.95		38.96-43.88		43.89-51.23		51.24-100	
	202	*542*	*241*	*351*	*378*	*414*	*373*	*268*	*326*	*220*	*441*	*208*	*486*	*218*	*576*	*163*	*579*	*111*	*905*	*114*
**Hsa-Dme**	0-20.99		21.00-25.45		25.46-28.78		28.79-31.68		31.69-34.62		34.63-37.94		37.95-42.00		42.01-47.10		47.11-55.16		55.17-100	
	205	*712*	*297*	*572*	*318*	*428*	*368*	*271*	*399*	*251*	*504*	*257*	*617*	*212*	*698*	*175*	*801*	*125*	*1095*	*143*
**Hsa-Dre**	0-32.55		32.56-39.39		39.40-45.10		45.11-50.13		50.14-55.17		55.18-59.94		59.95-65.17		65.18-70.71		70.72-78.19		78.20-100	
	331	*2058*	*424*	*952*	*542*	*779*	*661*	*765*	*885*	*661*	*955*	*601*	*1173*	*581*	*1223*	*448*	*1597*	*422*	*2108*	*379*
**Hsa-Gga**	0-36.92		36.93-44.24		44.25-50.16		50.17-55.54		55.55-60.46		60.47-65.37		65.38-70.29		70.30-75.71		75.72-82.10		82.11-100	
	238	*2942*	*332*	*1171*	*447*	*907*	*558*	*830*	*702*	*643*	*885*	*562*	*1019*	*470*	*1248*	*372*	*1637*	*266*	*4015*	*240*
**Hsa-Mmu**	0-45.79		45.80-52.66		52.67-58.14		58.15-62.76		62.77-66.98		66.99-70.97		70.98-75.13		75.14-79.45		79.46-85.04		85.05-100	
	175	*5824*	*211*	*1525*	*259*	*1115*	*342*	*833*	*458*	*670*	*615*	*486*	*876*	*371*	*1170*	*299*	*2200*	*277*	*9003*	*347*

[a] ortholog-ortholog pair

[b] ortholog-closest non-ortholog pair
