# Supplementary material for: Orthology confers intron position conservation
Source: BMC Genomics. 2010 Jul 2;11:412. doi: 10.1186/1471-2164-11-412 (PMC2996940; doi:10.1186/1471-2164-11-412)
Supplement: Additional file 4 — Bin boundaries and number of pairs in the different bins. Sequence identity bin boundaries and number of inparalog-inparalog (i-i) and inparalog-closest non-inparalog (i-cni) pairs in the different bins. The structure of data in each bin is given above the table. [file 1471-2164-11-412-S4.PDF]

Table S2. Sequence identity bin boundaries and number of inparalog-inparalog (i-i) and inparalog-closest non-inparalog (i-cni) pairs in the different bins. The structure of data in each bin is given above the table.

Bin #	
% sequence identity bin boundaries	
#i-i[a] pairs	#i-cni[b] pairs

	Bin #1		Bin #2		Bin #3		Bin #4		Bin #5		Bin #6		Bin #7		Bin #8		Bin #9		Bin #10	
**Hsa-Ath**	0-30.09		30.10-35.74		35.75-40.02		40.03-44.01		44.02-48.53		48.54-54.00		54.01-59.00		59.01-64.65		64.66-72.83		72.84-100	
	36	*523*	*54*	*180*	*70*	*110*	*80*	*81*	*99*	*55*	*172*	*57*	*138*	*30*	*169*	*26*	*208*	*25*	*706*	*20*
**Hsa-Cel**	0-29.15		29.16-34.09		34.10-38.20		38.21-41.69		41.70-45.32		45.33-49.58		49.59-54.41		54.42-60.66		60.67-70.30		70.31-100	
	33	*603*	*45*	*196*	*87*	*140*	*95*	*103*	*120*	*77*	*163*	*67*	*215*	*55*	*271*	*32*	*350*	*50*	*580*	*43*
**Hsa-Dme**	0-29.66		29.67-34.81		34.82-38.62		38.63-42.47		42.48-46.00		46.01-50.18		50.19-55.24		55.25-61.60		61.61-71.83		71.84-100	
	27	*634*	*59*	*208*	*60*	*125*	*103*	*128*	*109*	*76*	*181*	*63*	*237*	*58*	*304*	*43*	*382*	*57*	*565*	*50*
**Hsa-Dre**	0-37.69		37.70-45.78		45.79-51.78		51.79-57.50		57.51-62.91		62.92-68.69		68.70-74.76		74.77-81.71		81.72-87.45		87.46-100	
	35	*492*	*65*	*216*	*89*	*157*	*83*	*149*	*111*	*94*	*158*	*102*	*170*	*75*	*224*	*61*	*165*	*22*	*653*	*57*
**Hsa-Gga**	0-41.11		41.12-47.49		47.50-54.62		54.63-61.51		61.52-67.32		67.33-72.60		72.61-76.34		76.35-82.84		82.85-89.87		89.88-100	
	13	*146*	*11*	*39*	*17*	*50*	*32*	*35*	*31*	*12*	*27*	*13*	*18*	*10*	*40*	*15*	*56*	*10*	*225*	*20*
**Hsa-Mmu**	0-50.73		50.74-58.26		58.27-63.43		63.44-68.45		68.46-73.14		73.15-77.85		77.86-82.26		82.27-87.09		87.10-94.62		94.63-100	
	14	*307*	*9*	*59*	*9*	*41*	*18*	*37*	*22*	*30*	*29*	*26*	*48*	*20*	*66*	*17*	*127*	*24*	*424*	*34*

[a] inparalog-inparalog pair

[b] inparalog-closest non-inparalog pair
