# Supplementary material for: Orthology confers intron position conservation
Source: BMC Genomics. 2010 Jul 2;11:412. doi: 10.1186/1471-2164-11-412 (PMC2996940; doi:10.1186/1471-2164-11-412)
Supplement: Additional file 5 — Intron position conservation versus sequence identity. Intron position conservation scores for pairs of the different types binned according to their sequence identity shown for human versus four other species. Ortholog-ortholog (o-o) pairs versus ortholog-closest non-ortholog (o-cno) pairs, and inparalog-inparalog (i-i) pairs versus inparalog-closest non-inparalog (i-cni) pairs for (A) Hsa versus Cel, (B) Hsa versus Dme, (C) Hsa versus Gga, and (D) Hsa versus Mmu. [file 1471-2164-11-412-S5.PDF]

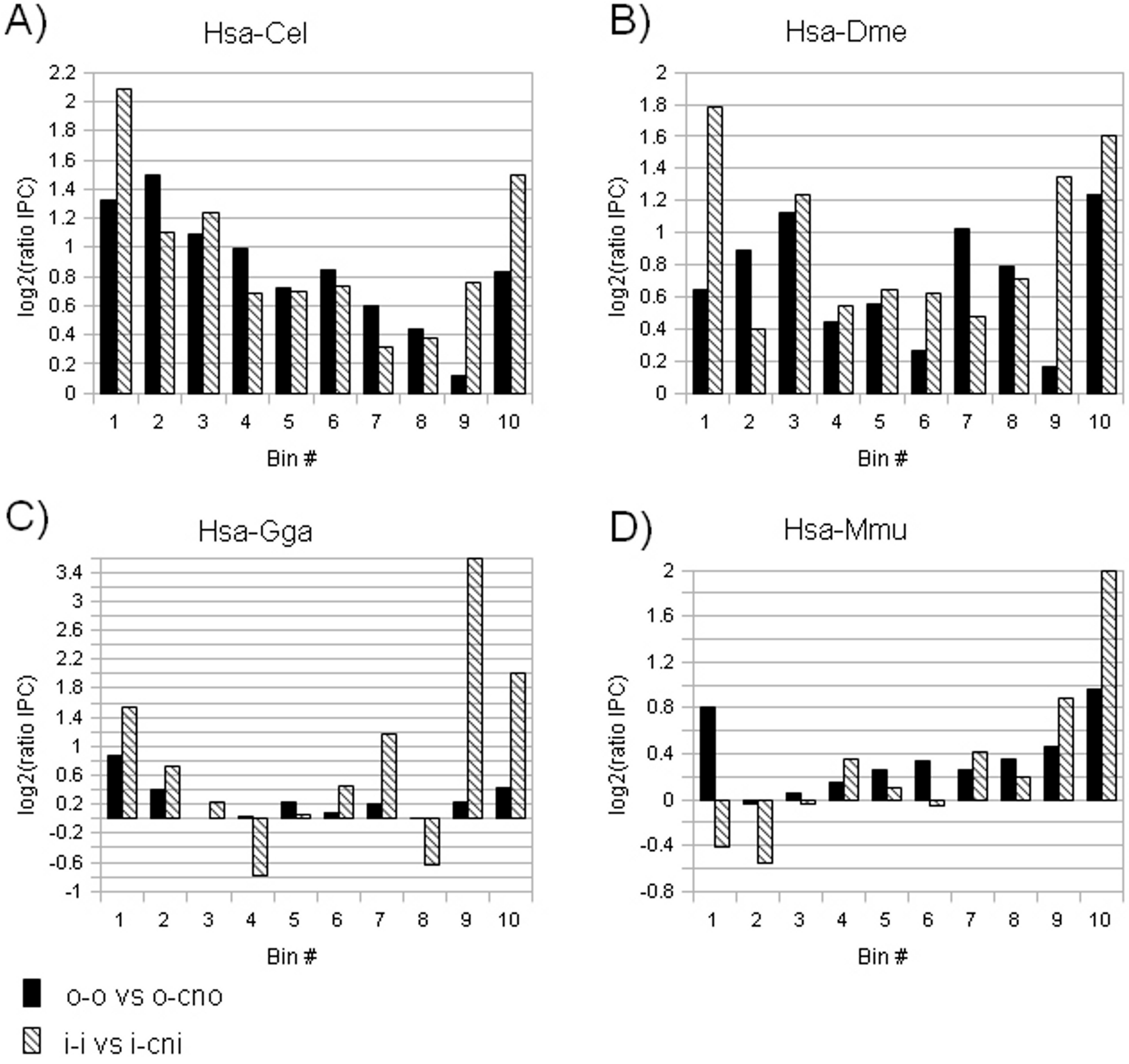

Figure S3. Intron position conservation scores for pairs of the different types binned according to their sequence identity shown for human versus four other species. Ortholog-ortholog (o-o) pairs versus ortholog-closest non-ortholog (o-cno) pairs, and inparalog-inparalog (i-i) pairs versus inparalog-closest non-inparalog (i-cni) pairs for (A) Hsa versus Cel, (B) Hsa versus Dme, (C) Hsa versus Gga, and (D) Hsa versus Mmu.
